# Behavioural profile and human adaptation of survivors after radical cystectomy and ileal conduit

**DOI:** 10.1186/1477-7525-12-46

**Published:** 2014-04-07

**Authors:** Maria Angela Cerruto, Carolina D’Elia, Giovanni Cacciamani, Davide De Marchi, Salvatore Siracusano, Massimo Iafrate, Mauro Niero, Cristina Lonardi, Pierfrancesco Bassi, Emanuele Belgrano, Ciro Imbimbo, Marco Racioppi, Renato Talamini, Stefano Ciciliato, Laura Toffoli, Michele Rizzo, Francesco Visalli, Paolo Verze, Walter Artibani

**Affiliations:** 1 Urology Clinic, University of Verona, P.zza L.A. Scuro 10, Verona 37134, Italy; 2 Urology Clinic, University of Trieste, Trieste, Italy; 3 Urology Clinic, University of Padua, Padua, Italy; 4 Department Time Space Image Society, Sociology section, University of Verona, Verona, Italy; 5 Urology Clinic, Catholic University of the Sacred Heart, Rome, Italy; 6 Department of Urology, University of Naples, Corso Umberto I, 40, Napoli 80138, Italy; 7 Unit of Epidemiology and Biostatistics, IRCCS-CRO Aviano, Aviano, Italy

**Keywords:** Radical cystectomy, Ileal conduit, Health related quality of life

## Abstract

**Background:**

There is a lack of good data in the literature evaluating the Health-Related Quality of Life (HR- QoL) in patients with urinary diversions. The aim of this study was to examine the changes in expectation and needs in terms of human adaptation and behavioural profiles in patients with ileal conduit (IC) after radical cystectomy (RC) for bladder cancer (BC).

**Materials and methods:**

A qualitative, multicenter cross-sectional study using a “narrative based” approach was planned. We proceed with a sampling reasoned choice (*purposive*), selecting groups of patients with follow-up from one up to more than 7 years after surgery. Data were collected through individual interviews.

**Results:**

Thirty patients participated in the study. The processing of the interviews allowed us to identify 2 major profiles: *positive* and *negative*. Patients with a *positive* profile resumed normal daily activities with no or limited restrictions both on the personal and the social level. This profile reflects a good HR-QoL. The *negative* profile reflects the patients for whom the ostomy has meant a worsening of HR-QoL. A *positive* profile was statistically more frequent in older patients (p = 0.023), with a longer follow-up (p = 0.042) and less complications rates (p = 0.0002). According to the length of follow-up and the occurrence of complitations, we identified further 5 intermediate profiles.

**Conclusions:**

Patients’ satisfaction is related to the degree of adaptation to their new life with an urinary stoma and its correct management. Live “with urinary diversion” represents a new phase of life and not a deterioration.

## Introduction

Bladder Cancer (BC) is the most common malignancy of the urinary tract and the seventh most common cancer in men and the 17th in women [1].

In the literature, several surgical options of urinary diversion after RC have been described, from simply diverting the urine through a conduit to orthotopic reconstruction. The ideal urinary diversion after RC should be easy to prepare and easy to handle, presenting few complications, low mortality and morbidity; moreover it should protect the upper urinary tract function and should be well accepted by the patient, thereby ensuring the best Health-Related Quality of Life (HR-QoL) as possible.

From the study of the literature on the impact of the different urinary diversions on patients’ HR- QoL after RC, there were no significant differences mainly because of the generic tools of assessment used for the analysis, not suitable and unreliable for this purpose and unable to specifically measure the HR-QoL in this patients population [2,3]. The main issue is that in clinical practice we have not suitable tools to meet patients’ needs along their illness trajectory because we do not know them. Recently Mohamed et al. [4] tried to fill this lack of information identifying many patients’ unmet needs along their illness course. These authors highlighted the importance to meet all patients’ needs to ensure patients adequate involvment in their healthcare and to enhance post-surgical HR-QoL. They concluded that because patients’ informational and support needs vary along the cancer trajectory, an effective support provision plan should follow changes in patients’ need.

In order to obtain a clear description of the evolution of the needs and expectations of the patients over the time, we planned a qualitative, multicenter study evaluating the adapting strategies of patients undergone RC and IC along their illness course using a “narrative based” approach [5-7], identifying specific behavioural profiles according to the follow-up.

## Materials and methods

This cross sectional study involved 3 urological academic centres (PD, TR, VR). Because in qualitative research, sampling is not probabilistic, we proceeded with a sampling reasoned choice (*purposive*); thus the units were selected from those who were thought to be more related to the phenomenon under study. In other terms we identified, on the basis of the clinical literature, periods of follow-up in which the patient HR-QOL was likely to vary. Then we selected groups of patients undergoing RC and IC with a follow-up from one year up to more than 7 years after surgery. This procedure is similar to that of the stratification in the statistical sampling, although the extraction of the cases has not been operated with randomized criteria nor determining the size of the sample on the basis of the sampling error. By contrast, in qualitative research the principle that guides the procedure is the saturation of information [8,9]. According to this principle, the optimum sample size would be determined during the study itself, when redundant information may emerge from the empirical survey, in other words, when the addition of a further case (to those already detected) would not add more information.

In selecting patients the following inclusion criteria were considered:

– patients undergoing RC and IC;

– both genders;

– aged between 18 and 80 years;

– educational level sufficient to understand and fill out a questionnaire;

– sufficient command of the Italian language;

– absence of cognitive impairment.

We excluded all patients with: a history of psychiatric disorders, alcohol and/or substance abuse, difficulties in verbal and/or written communication. All patients provided written informed consent. The study was approved by the Ethics Committees of Verona and Rovigo (protocol number 20097339KC).

### Qualitative study protocol

The qualitative study was characterized by a biographical approach [8,9] guided by the principles of Grounded Theory [10,11] in order to assess differences between patients in the different subgroups of follow-up and possible changes of their expectations over time. All aspects considered most important for the HR-QOL over time have been investigated, such as those relating to the “*body image*”, autonomy in the management of the derivation and any change according to the length of follow-up.

The above mentioned approach applied to the evolving needs and expectations in a small number of patients, selected to represent the various phenomena of their state, has been, therefore, considered the most suitable for our purpose and a qualitative and narrative based interview granted the necessary process of listening to the patient through a very refined technique of conversation that leads the interviewer to understand the actual experience of the patient, through listening to interviewer and patient emotions [5-7].

The Interview Guide (IG) implemented at this stage aimed at the preliminary qualitative study of HR-QoL to the different timing of follow-up, considering the history of disease narrated by the patient and taking advantage of the changes both in their own way of living with an ostomy, and in the evolution of expectations from the surgery until the last follow-up.

The interview guide was constructed, in order to investigate the major changes of the perceived quality of life of patients. For each theme proposed, the alterations occurred immediately after surgery, time after surgery and finally time compared to today, at the time of the interview, are investigated. The main topics contained in the IG were: concerns before surgery, complications after surgery, changes in daily life (expected and unexpected), problems with management of the stoma (adopted solutions, habits with the ostomy), problems at work (with colleagues, with the employer, possible desertion due to the stoma), family, marital and friendship relationships, (isolation, stigma, renounce to movement and to friendships) recalibration of its own scale of values [12], changes in health and well-being or not perceptions.

All patients were first contacted by telephone. During the first phone contact we explained the reason for calling asking the patients to go to their urological reference centre for a face-to-face interview. All patients signed an informed consent. The interviews took place in a dedicated clinic to maintain the privacy of patients.

Interviewers set up a dialogue with the patients, asking them to describe their experience so that the answers to the questions were as open as possible. The purpose of this phase was to obtain a clear description of the evolution of the needs and expectations of the patients over the time.

### Statistical analysis

The t-test, Mann–Whitney and Pearson Chi-square were used to examine patients’ profiles differences according to the following demographic and clinical features: gender, age, recruitment center, follow-up, post-surgical complications. Statistical analysis was performed using SPSS v. 20.0 (IBM Corp, Armoni, NY, USA). A 2-sided p < 0.05 was considered statistically significant.

## Results

We interviewed a total of 30 patients who underwent RC with IC at a mean follow-up of 7 years (± 63.07), with a mean age of 76.43 years (± 7:41); 13 were females (mean age 77.15 ± 8:47) and 17 males (mean age 75.88 ± 6.71) (Table 1). The overall long-term complication rate was 33.33%. The processing of the interviews allowed us to identify 2 major profiles grounded through the content analysis [13] of the interviews:

– *Positive*: patients who reported having a good management of the ostomy and good relations with it belong to this profile. These patients resumed normal daily activities with no or limited restrictions both on the personal and the social level. This profile reflects a good QoL. *- Negative:* this profile reflects the patients for whom the ostomy has meant a worsening of QoL. This profile is characterized by a difficult management of the ostomy and a problematic coexistence with it, or anything associated with a partial resumption of daily activities.

**Table 1:** Patients’ characteristics

**Patient progressive ID**	**Gender**	**Age (years)**	**Follow-up (years)**	**Recruitment centre**	**Post-surgical complications**
1	Male	65	8	VR	YES
2	Male	78	8	VR	NO
3	Male	71	8	VR	NO
4	Male	84	8	VR	NO
5	Male	67	4	VR	YES
6	Male	67	4	VR	YES
7	Female	65	10	VR	YES
8	Male	81	2	VR	NO
9	Male	77	5	VR	NO
10	Female	85	7	VR	NO
11	Female	72	10	PD	NO
12	Female	88	3	PD	YES
13	Female	72	4	PD	YES
14	Female	73	3	PD	YES
15	Female	79	16	PD	NO
16	Female	79	3	PD	YES
17	Female	81	3	PD	YES
18	Male	79	1	PD	NO
19	Male	79	3	PD	NO
20	Male	89	30	PD	NO
21	Female	76	7	PD	NO
22	Female	94	21	PD	NO
23	Female	65	2	PD	NO
24	Male	69	4	PD	YES
25	Male	70	7	TR	NO
26	Male	81	3	TR	NO
27	Female	74	5	TR	NO
28	Male	75	5	TR	NO
29	Male	80	6	TR	NO
30	Male	78	10	VR	NO

A *positive* profile was statistically more frequent in older patients (mean age 79.06 ± 6.46 vs 73.00 ± 7.38 years, p = 0.023), with a longer follow-up (8.94 ± 7.28 vs. 4.46 ± 2.43 years, p = 0.042) and less complications rates (11.76% vs 61.54%, p = 0.0002). According to the length of follow-up and the occurrence of complications, we identified 5 intermediate profiles, such as:

– *1-year intermediate profile* (1-2-year follow-up): for some patients at 1 year after surgery QoL is definitely worse compared to the pre-intervention condition (poor sleep, difficult to manage the ostomy, dependent on other people). However, for other patients the perception of his/her own life in terms of QoL is not changed after surgery.

– *3-year intermediate profile* (3-4-year follow-up): this profile is characterized by a dramatically negative perception of QoL. Although for some patients QoL remains unchanged, the majority of patients feels a 100% worsening of their QoL with severe limitiations in their daily activities. The main factors responsible for this drastic worsening are: dependence on partners in the management of the ostomy (change and cleaning); the strong and constant concerns about urinary stoma leaks, frequent unintentional plate detachment, urine smell; lack of sexual relationships; giving up to do social activities or to go out (beause of the fear of the risk of urine leakage and unintentional detachment of the base plate).

– *5-year intermediate profile* (5-6-year follow-up): Five years after surgery patients’ perception of QoL improves. Most of the patients considered the QoL satisfactory or good. It is reported a greater degree of independence (or less dependent) by the partner in the management of the urinary stoma. The fear of an unintentional detachment of the plate remains but over time the patients learned to prevent this adverse event (e.g. more frequent bag changes). Friendship and social interactions are enough. At the time of the interview they felt a better state of health than before surgery even in the presence of an increased fatigue (mainly due to aging in most cases).

– *7-year intermediate profile* (7-8-year follow-up): Seven years after surgery patients’ lifestyle changes. They do not depend on urinary stoma anymore. The quality of life becomes good, as well as the state of health. Some patients report that it is as if they had never made the ostomy. The recovery of social relations improves their QoL significantly. Partner’ help in the management of the urinary stoma is less dramatic and although it is always present, the degree of dependence becomes less and less relevant. Lack of sexual activity and fear for the plate detachment still remain. Possible problems arise from long-term complications (such as stoma hernias, urinary tract infection, stoma obstruction, bleeding or vescicles) rather than from the urostomy itself. Patients perceived a better state of health than before surgery, less concern about their health related to the stoma and greater peace of mind related to the operation outcomes.

– *>7-year intermediate profile* (from 10 up to 30-year follow-up): The long coexistence with an ostomy has changed the attitude of patients towards it. It is part of themselves. Patients no longer fear plate detachment. They do not feel any daily activity limitation. The only concerns derive from possible long-term complications. They describe a long established practice for the management of the stoma (cleaning and change). Patients do not feel fully dependent on partners, but both work together for the correct management of the urinary stoma. There remain concerns about the gap, exacerbated by problems such as hernias that worsen the adhesion of plaque to the skin. There remains the absence of sexual relations. Lack of sexual activity still persists.

Table 2 includes examples of specific comments from patients’ interviews.

**Table 2:** Specific comments from patients’ interviews

**3 year profile**	**5 year profile**	**7 year profile**	**+7 year profile**
I broke up my friendships […] I don’t go out to dinner anymore, I don’t feel confident about myself. If something happens, I have to go home if I don’t find the right condition to dress me up (man, 67 years old, four years after surgery, retired because he quit his job)	I feel really fine, I go to gym, to walk […] my quality of life is very good […] Now I’m healed (male, 75 years old, 5 years after surgery, retired)	At first I had erection problems, but after it was all ok […] nothing has changed with my friends, my relationships are even better […] (male, 70 years old, 7 years after surgery, retired)	If I think back to the ostomy I feel good. Today I have other problems. The old age […] (male, 89 years old, 30 years after surgery, retired)
I’m terrified of this thing [that stoma disconnects] I don’t feel pain, but I have this fear, it is not easy to live in such way […] I feel sluggish and tired and it takes me half the day to make the bed […] is a great trauma (Woman, 81 years old, 3 years after surgery, retired)	At the time I don’t have anxiety about my condition, there are some precautions that should be observed […] I must be careful that there are no leaks, but it happens rarely in my case and I can live almost normally (male, 80 years old, 6 years after surgery, retired)	I didn’t expected, that they told me, that I couldn’t have sex […] there were some concerns and even now sometimes I complain of plate disconnection, but it’s a normal thing … It’s just like I have not undergone this operation, I eat, drink and have fun (male, 71 years old, 8 years after surgery)	I feel better now, I was better after the operation, my problem today is the stroke, but yes, before I was better (female, 79 years old, 16 years after surgery, retired)
Earlier I used to go to the gym, after the operation I didn’t training anymore. I feel more tired […] now I have the hobby of embroidery […]	The relationship with my wife has become deeper and we try to live intensely our last few years, we travel some more, we try to live as best as we can […] perhaps sex life was important and I was sorry for my wife … (male, 75 years, 5 years after surgery, retired)	Now, sexual relationships, much less than before… They told me, but it was a bit tragic […] (male, 65 years old, 8 years after surgery)	[My health today is] appropriate to my age. I judge it well. I do not have a disease today … I just want to be able to get up by myself, to walk without staggering … well, this makes me angry … (Woman, 94 years, 21 years after surgery, retired)
Earlier I was on journey oft, now no more […] I miss it, I liked this escapism, it gave me a boost… (woman, 88 years old, 3 years after surgery, retired)			
I don’t have more friends, no more sex life, I feel destroyed, physically, emotionally, once I was master of myself, now I depend on my wife … The surgery carried away all that I had (male, 81 years old, 3 years after surgery, retired)		I’ve never had problems with the plate, today my biggest problem is the hernia, probably some internal stitch broke […] apart from that I’m fine, I tell the truth (male, 78 years, 8 years after surgery, retired)	[My quality of life today is] good because before [the operation] was hard to do anything. The way of life has not changed, because the job did not give limitations on me, I have travelled and I hope to continue, I have contacts with people and I’m part of a voluntary association … I think it’s a good way of life (woman, 72 years, 10 years after surgery, retired)
		I clean, she (his wife) puts the plate when I’m lying down, and centres it good (male, 71 years old, 8 years after surgery)	
		(There is) a problem of adherences. Then they “attached” two canulas to ureters and they should be changed every two months, with monthly antibiotic treatment. The quality of life is poor. We are always in the hospital (Woman, 76 years, 7 years after surgery)	

## Discussion

This is the first qualitative study evaluating a cohort of patients at progressive and different follow- up, making it possible to highlight the changes in patients’ expectation and needs as they learn to manage the urinary stoma and to live with it. The main aim of this qualitative study was to select the most appropriate tools to get “specific” rather than “generic” answers in the assessment of QoL in patients who underwent radical cystectomy and IC. The processing of the interviews allowed us to identify 2 major profiles (“*positive”* and “*negative”*) and 5 intermediate profiles. The 1st year after surgery is characterised by the cohesistence of the two opposite and homogeneous situations described as *positive* and *negative*. However, during the 1st year after surgery, the experience perceived by the patients is better in terms of QoL compared to that perceived in the 3rd year after surgery. This finding is due to the fact that within the 1st year after cystectomy patients are optimistic about their treatment made and recovery. After 3 years of follow-up, patients begin to take stock of their situation and the comparison between their current situation and the life before cancer is inevitable. This greatly affects patients’ experience, who feel trapped in a new situation strongly dependent on urinary diversion. Patients’ life completely changes, and the feeling of not to being the same as before takes precedence over all aspects of QoL: Since the 5th years after surgery the *positive* profile becomes more and more frequent. This change in perspective is due to the phenomenon of adaptation. Actually according to Canguilhem’s definition, health is not something defined statistically or mechanistically, but rather as the ability to adapt to one’s environment [14]. Health is not a fixed entity. It varies for every individual, depending on their circumstances. Health is defined not by the physician, but by the person, according to his or her functional needs. The role of the physician is to help the individual adapt to their unique prevailing conditions. Which comes fairly close to defining “personalised medicine” [14]. Canguilhem’s definition of health, or rather of normality, includes the animate and inanimate environment, as well as the physical, mental, and social dimensions of human life. Therefore it puts the individual patient in a position of self-determining authority to define his or her health needs. Through this definition, healthcare gets a place in the equation as the doctor (or other healthcare worker) becomes a partner in delivering care according to the needs of the patient. Thus making healthcare a service delivered to the individual in need of care. It ‘obvious that the adaptation to the new condition after cystectomy has a significant role in the evaluation of patients’ QoL.

Recently Mohamed et al. [4] highlighted the importance to know all patients’ needs in order to ensure patients’adequate involvement in their healthcare, and to enhance post-surgery QoL. Using our narrative approach we might observe that initially the urinary diversion is not accepted or it perceived as a limiting factor, over time it becomes an aspect of patient’s life. The daily activities are no longer influenced by the presence of the urinary stoma. The ostomy management becomes more and more familiar and the patients, although they always need the support of their partners, are no longer completely dependent. The recovery of relational activities plays a very important role in QoL, significantly improving the patient’s experience. Although since the third year of follow-up QoL improves significantly in most cases, some aspects still remain critical. The continuous worrying about the unintentional detachment of the urinary stoma plate or bag leads patients to live in a constant state of alert in order to cover such eventuality. In some cases, this fear restricts patients conditioning their social relations. Although episodes of detachment are drastically reduced over time because of a better and consolidated management of the external urine collection device, the fear of a possible unintentional detachment still remains. Another aspect that may affect patients’ QoL in the long term is the concern about losing their partners, who not only help them in the management of the stoma, but they represent a moral support of paramount importance.

Another aspect reported by the patients even after many years after surgery is the renunciation of even short trips because of the fear of losing urine. Many patients must be sure to always have everything they need for a proper management of the ostomy in any situation.

A further aspect that may negatively affect patients’ QoL, mainly in the long term, is the presence of late postoperative complications such as hernias, urinary tract infections, stenosis of the urinary stoma, peristomal skin lesions, prolapse of the stoma and the weight gain, able to worsen the ostomy management.

In 2010 Gemmill et al. evaluated the health-related quality of life among cancer patients with continent and incontinent urinary diversion, mailing to 2890 individuals the City of Hope HRQOL-Ostomy Questionnaire. Of the 1,600 returns, there were 307 responses from patients with urinary diversion. Less than 27% of prior sexually active patients resumed sexual activity after surgery, while more than 40% of patients reported feeling depressed, and most patients also reported difficulty in adjusting to their diversion, difficulty with urine leakage, skin problems, difficulties in managing care, fear of recurrence, financial worries, family distress, and uncertainty about the future [15].

Also Richbourg et al. evaluated the difficulties related to the presence of a ostomy. The colleagues mailed 140 surveys to ostomates who are 18 years older and who have undergone a urinary or fecal diversion. The surveys demonstrated a return rate of 31%; the more frequent difficulties referred by the patients were, as in the forementioned study: peristomal skin irritation (76%), pouch leakage (62%), odor (59%), reduction in previously enjoyed activities (54%), and depression/anxiety (53%).

It’s important to observe, moreover, that about 20% of the patients did not seek help. However, even if the concerns referred by the patients are overlapping, our study if the first qualitative study evaluating a cohort of patients at progressive and different follow-up, highlighting the changes in patients’ expectation, needs and apprehensions during the time [16].

With regard to rectal cancer patients, in 2013 Recalla et al. published a sistematic review, evaluating the ostomy care management for colostomy, ileostomy and urostomy, as a part of a best practice guideline document generated by a task force appointed by the Registered Nurses’ Association of Ontario. All the 32 evaluated studies was quantitative studies. Generally, the studies reported that patients with stoma experienced poorer results in emotional, social, cognitive functioning, low self esteem and depression, but long term qualilty of life, when compared to the general population, mirrors that of the normal population [17].

Recently, Beaver et colleagues evaluated patient perceptions of follow-up care in patients after treatment for colorectal cancer. The dominant theme was ‘knowing what to expect’ after bowel surgery, and the authors concluded that nurse-led clinics and/or telephone follow-up by specialist nurses may be effective models of care for this particular patient group, providing appropriate access for meeting clinical, psycho-social and information needs. Although this study focused the attention on patients with ostomy after colorectal cancer treatment, the patients’ worries are oriented on the future life’s expectations, as in our case. The authors concluded that traditional follow up methods of routine hospital follow-up may be effective on disease recurrence, but does not always address patients’ psycho-social and information needs [18].

Moreover, innovative strategies of follow up are needed, introducing qualitative approaches such, for instance, our interviews.

## Conclusions

Patients’ satisfaction is related to the degree of adaptation to their new life with an urinary stoma and its correct management. Live “with urinary diversion” represents a new phase of life and not a deterioration.

## Abbreviations

BC: Bladder cancer; HR- QoL: Health-related quality of life; IC: Ileal conduit; IG: Interview guide; RC: Radical cistectomy.

## Competing interests

The authors declare that they have no competing interests.

## Authors’ contributions

Study concept and design: MAC, CL, MN. Acquisition of data: MAC, CD’E, GC, DDM, SS, MI, MN, CL, PB, EB, CI, MR, RT, SC, LT, MR, FV, PV, WA. Analysis and interpretation of data: MAC, CL. Drafting of the manuscript: MAC, CL, MN. Critical revision of the manuscript for important intellectual content: MAC, WA, CL, MN. Statistical analysis: MAC. Supervision: MAC, CL, MN, WA. All authors read and approved the final manuscript.
